# To the Physicians and Meteorological Observers of the Valley of the Mississippi and the Lakes

**Published:** 1845-12

**Authors:** Daniel Drake

**Affiliations:** Medical Institute of Louisville


// THE WESTERN JOURNAL.

NEW SERIES.—VOL. IV.—NO. VI.

LOUISVILLE, DECEMBER 1, 1845.

TO THE PHYSICIANS AND METEOROLOGICAL OBSERVERS OF THE VALLEY OF THE MISSISSIPPI AND THE LAKES.

The undersigned, having at last become engaged in the composition of a systematic treatise on the physical condition and diseases of the region between the Gulf of Mexico and the Northern Lakes, for which he has been collecting materials by travel, respectfully requests the physicians and scientific gentlemen, who, in his personal interviews with them, promised to assist him with facts, to forward them, by mail at his expense, or by private opportunity. Post mortem examinations, remarkable cases, and histories of epidemics of all kinds, will be acceptable. In short, any well observed and well recorded fact, going to illustrate the causes, symptoms, or treatment of any of our diseases, will be gratefully received. As the morbid appearances in those who have died of our autumnal fevers, have been but imperfectly set forth, in any published accounts, he is extremely desirous of receiving cases of dissection of that kind. He is likewise anxious to obtain accounts of the typhoid fever, and the erysipelatous fever, which, for the last few years, have prevailed in various parts of the West and South.

As he will aim at giving a fuller account of the climate of this region than has hitherto been published, he asks for meteorological observations, from all observers, both in and out of the profession. Those who have published abstracts, will please to send them, and those who have not, will greatly oblige him, and give additional value to their labors, by furnishing him with manuscript tabular views, presenting monthly extremes and means of temperature and pressure; the courses of the winds reduced to eight points of the compass; the proportion of clear and cloudy days; the dew point, and the quantity of rain.

He would also be pleased to receive an accurate notice of the times, in the ensuing spring, when the following plants begin to unfold their leaves and flowers, and when the latter are full blown:—The peach tree, *(Amygdalus Persica)*—white flowering locust, *(Robinia pseudacacia)*—dogwood, *(Cornus Florida)*—and cotton wood, *(Populas angulata.)* In every case a number of individual trees should be observed, and the aspect of the spot, in reference to the sun, recorded. He wishes the meteorological observations to come down to the end of the present year, 1845—those of a later date he would not be able to use.

As to the time when his work will be ready for the press, the undersigned cannot at present speak with certainty; but all the causes which prevented his laboring upon it, having been overcome, and considerable progress in its composition made, he begins to look forward to its completion at no distant day.

That this notice may the more effectually reach those to whom it is directed, the undersigned would respectfully ask its insertion in those medical Journals which exchange with the Western.

DANIEL DRAKE, M.D.

*Medical Institute of Louisville,*
December 1st, 1845.

